# Estrogen receptor negative/progesterone receptor positive breast cancer is not a reproducible subtype

**DOI:** 10.1186/bcr3462

**Published:** 2013-08-23

**Authors:** Marco M Hefti, Rong Hu, Nicholas W Knoblauch, Laura C Collins, Benjamin Haibe-Kains, Rulla M Tamimi, Andrew H Beck

**Affiliations:** 1 Department of Pathology, Beth Israel Deaconess Medical Center and Harvard Medical School, Boston, MA, USA; 2 Channing Division of Network Medicine, Department of Medicine, Brigham and Women’s Hospital and Harvard Medical School, Boston, MA, USA; 3 Integrative Systems Biology, Institut de Recherches Cliniques de Montréal, University of Montreal, Montreal, QC, Canada

**Keywords:** Estrogen receptor, Progesterone receptor, Breast cancer, Immunohistochemistry, Gene expression microarrays, Biomarkers, Inter-assay reproducibility

## Abstract

**Introduction:**

Estrogen receptor (ER) and progesterone receptor (PR) testing are performed in the evaluation of breast cancer. While the clinical utility of ER as a predictive biomarker to identify patients likely to benefit from hormonal therapy is well-established, the added value of PR is less well-defined. The primary goals of our study were to assess the distribution, inter-assay reproducibility, and prognostic significance of breast cancer subtypes defined by patterns of ER and PR expression.

**Methods:**

We integrated gene expression microarray (GEM) and clinico-pathologic data from 20 published studies to determine the frequency (n = 4,111) and inter-assay reproducibility (n = 1,752) of ER/PR subtypes (ER+/PR+, ER+/PR-, ER-/PR-, ER-/PR+). To extend our findings, we utilized a cohort of patients from the Nurses’ Health Study (NHS) with ER/PR data recorded in the medical record and assessed on tissue microarrays (n = 2,011). In both datasets, we assessed the association of ER and PR expression with survival.

**Results:**

In a genome-wide analysis, progesterone receptor was among the least variable genes in ER- breast cancer. The ER-/PR+ subtype was rare (approximately 1 to 4%) and showed no significant reproducibility (Kappa = 0.02 and 0.06, in the GEM and NHS datasets, respectively). The vast majority of patients classified as ER-/PR+ in the medical record (97% and 94%, in the GEM and NHS datasets) were re-classified by a second method. In the GEM dataset (n = 2,731), progesterone receptor mRNA expression was associated with prognosis in ER+ breast cancer (adjusted *P* <0.001), but not in ER- breast cancer (adjusted *P* = 0.21). PR protein expression did not contribute significant prognostic information to multivariate models considering ER and other standard clinico-pathologic features in the GEM or NHS datasets.

**Conclusion:**

ER-/PR+ breast cancer is not a reproducible subtype. PR expression is not associated with prognosis in ER- breast cancer, and PR does not contribute significant independent prognostic information to multivariate models considering ER and other standard clinico-pathologic factors. Given that PR provides no clinically actionable information in ER+ breast cancer, these findings question the utility of routine PR testing in breast cancer.

## Introduction

Evaluation of hormone receptor expression is a central component of the pathological evaluation of breast cancer [1]. The biologic, prognostic and predictive importance of assessment of estrogen receptor (ER) expression in breast cancer is well established; however, the added value of progesterone receptor (PR) assessment is controversial [2-4]. Despite this uncertainty, the American Society of Clinical Oncology and the College of American Pathologists recommend testing for both ER and PR on all newly diagnosed cases of invasive breast cancer [1].

Since the 1970s, it has been hypothesized that PR expression will be associated with response to hormonal therapies in ER+ breast cancer, as it is thought that ER and PR co-expression demonstrates a functionally intact estrogen response pathway [5-8]. Analyses from observational studies showed that loss of PR expression was associated with worse overall prognosis among ER+ breast cancers [9-13]. These results suggested that evaluation of PR status in ER+ breast cancer might be used to help guide clinical management, as high levels of PR expression may identify a subset of ER+ patients most likely to benefit from hormonal therapy [7].

However, a recent meta-analysis of long-term outcomes of 21,457 women with early stage breast cancer in 20 randomized trials of adjuvant tamoxifen identified ER expression as the sole pathological factor predictive of response with no significant independent contribution by PR (relative risk of recurrence following tamoxifen treatment as compared with placebo or observation was 0.63 (SE 0.03) in the ER+/PR+ group and 0.60 (SE 0.05) in the ER+/PR- group) [14]. These data show that although PR negativity is associated with a more aggressive subtype of ER+ breast cancer, evaluation of PR expression cannot be used to identify ER+ patient subsets most likely to benefit from hormonal therapy. Consequently, the clinical utility of PR evaluation in ER+ breast cancer is uncertain.

The biological and clinical significance of the ER-/PR+ breast cancer subtype is even more controversial, with some reports claiming it represents a distinct, clinically useful biologic entity [15,16], while others posit that ER-/PR+ classification is primarily a technical artifact [17,18] and too rare to be of clinical use [2]. In large published series, the percentage of ER-/PR+ cases has been in the range of zero [18] to four percent [11,19]. In the Early Breast Cancer Trialists’ Collaborative Group (EBCTCG) meta-analysis, PR expression was not significantly predictive of tamoxifen treatment response in ER-negative breast cancer, although there was a slight trend, which failed to reach statistical significance [14]. In the EBCTCG analysis, the investigators noted that as methods for assessment of hormone receptor status have improved, the proportion of cases reported as ER-/PR+ has decreased from approximately 4% in the early 1990s to only 1% in recent SEER (Surveillance, Epidemiology, and End Results) cancer registry data, suggesting that as methods of ER testing and interpretation have improved, the rates of false negative ER results have decreased [14]. Given the rarity and uncertain clinical and biological significance of the ER-/PR+ classification, it has been recommended that patients classified as ER-/PR+ should undergo repeat ER testing to rule out a false negative result [1].

**Figure 1:**
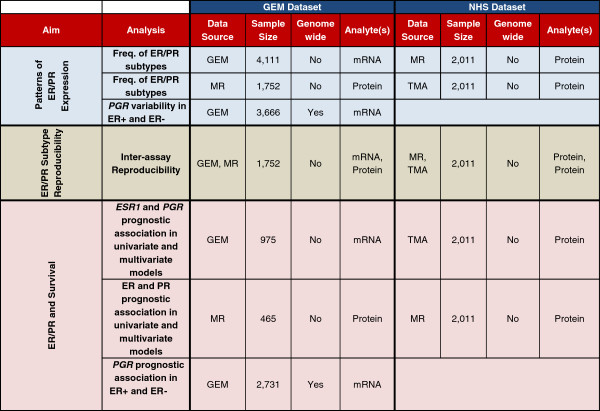
**Overview of study design and analyses performed.** MR, medical record; GEM, gene expression microarray; TMA, tissue microarray.

Thus, despite the fact that ER and PR evaluation have played central roles in breast cancer diagnostics and research since the 1970s, it is currently not well established if the joint assessment of ER and PR stratifies breast cancers into four biologically meaningful and clinically useful subgroups (ER+/PR+, ER+/PR-, ER-/PR-, and ER-/PR+). To provide further insight into the biology of ER and PR expression and the clinical utility of ER and PR testing in breast cancer, we performed an integrative analysis, incorporating gene expression profiling data, survival data and ER and PR protein expression data from several large cohorts of breast cancer patients (Figure 1).

The primary aims of our study are to:

1) Determine the frequency and reproducibility of breast cancer subtypes defined by ER and PR expression levels.

2) Determine the association of PR expression with survival in ER+ and ER- breast cancer and assess the contribution of PR to multivariate prognostic models, including ER and standard clinico-pathologic factors.

## Methods

### Study overview

An overview of the study design and the set of analyses performed on the GEM and NHS datasets are shown in Figure 1.

### Gene expression microarray (GEM) cohort

We integrated data from a total of 20 previously published gene expression microarray datasets. Nineteen of the datasets were initially provided as supporting material in [20], and the 20th dataset comes from The Cancer Genome Atlas (TCGA) breast cancer cohort [21]. To access the TCGA data, we downloaded the Level 3 loess normalized Agilent (Santa Clara, CA, USA) microarray mRNA expression data from the Broad Institute’s Genome Data Analysis Center. None of the public gene expression microarray data used in this study required additional consent to analyze or publish results obtained from the data. Further description of the datasets is provided in Additional file 1: Table S1.

### Gene expression profiling data scaling and merging

The datasets used in our study were generated using diverse microarray platforms and originating from different laboratories. We used normalized log_2_(intensity) for single-channel platforms and log_2_(ratio) in dual-channel platforms. Hybridization probes were mapped to Entrez Gene ID. When multiple probes mapped to the same GeneID, we used the probe with the highest variance in the dataset under study. We scaled and centered expression values for each gene to have a mean of zero and standard deviation of one in the dataset, prior to merging the data from the different datasets. The complete dataset contains data on 4,111 patients (all with ER and PR measurements). For the genome-wide analyses, we limited the analysis to the 3,666 patients with valid data from at least 80% of the genes.

### Estrogen receptor and progesterone receptor mRNA expression

We obtained gene expression profiling data on estrogen receptor (*ESR1*) and progesterone receptor (*PGR*) mRNA expression from 4,111 patients. Patients were classified as ER+/ER- and PR+/PR- by modeling a mixture of two Gaussians from the estrogen receptor mRNA and progesterone receptor mRNA expression levels (separately). This procedure was implemented with the Mclust function in the *mclust* package in R with equal variance. A similar approach to subtyping was used in [20]. After subtyping by *ESR1* and *PGR* mRNA expression separately, patients were classified into joint ER/PR categories: ER+/PR+, ER+/PR-neg, ER-/PR-neg and ER-/PR+.

### ER and PR protein expression in the GEM dataset

We obtained protein expression data from immunohistochemistry (IHC) from the clinical data provided in [20] and from the Broad Institute’s Genome Data Analysis Center for patients from TCGA. In total, we obtained matched mRNA and protein expression data for *ESR1*/ER and *PGR*/PR for 1,752 patients in the GEM dataset.

### Assessment of agreement between gene expression- and protein-based ER/PR classifications in the GEM dataset

To assess inter-assay reproducibility, we computed the proportion of cases in each diagnostic category as determined from the protein expression data in the medical record (MR) that were classified into the same diagnostic category using the mRNA expression data. For each binary diagnostic classification schema (ER+/PR+ vs. other; ER+/PR- vs. other; ER-/PR- vs. other; and ER-/PR+ vs. other), we computed Cohen’s Kappa statistic [22]. The Kappa score is widely used in studies of diagnostic agreement and interpretation can be aided by published guidelines: (<0 no agreement; 0 to 0.2 slight; 0.21 to 0.40 fair; 0.41 to 0.60 moderate; 0.61 to 0.80 substantial; 0.81 to 1 almost perfect) [23]. Kappa statistics were implemented in R using the Kappa function in the *vcd* package.

### Survival analyses in the GEM dataset

#### Univariate survival analysis of gene expression in ER+ and ER- breast cancer

We used the survival data and “traditional scaled” breast cancer gene expression profiling data for 2,731 patients and 13,091 genes provided in [24]. Patients were stratified into ER+ (n = 2,013, 74%) and ER- (n = 718, 26%) subtypes by modeling a mixture of two Gaussians from the *ESR1* mRNA expression levels. Univariate survival analyses were performed using the Cox Proportional Hazards model, implemented with the coxph function in the survival package in R. The statistical significance of each gene’s survival association was estimated based on the gene’s Wald Test *P*-value in the Cox model. Survival *P*-values were adjusted for multiple hypotheses using the method of Benjamini and Hochberg [25].

#### Multivariate survival analysis of ESR1 and PGR expression levels in breast cancer

We obtained mRNA expression data on *ESR1* and *PGR* expression and information on overall survival, age, grade, lymph node status and tumor size for 975 patients. We obtained information on ER and PR protein expression with overall survival, age, grade, lymph node status and tumor size for 465 patients. Using these data, we built multivariate Cox regression models to overall survival.

### Data visualization in the GEM dataset

For visualization of the high-dimensional data in our analyses, we produced smoothed versions of scatterplots with colors representative of the data densities. The smoothed scatterplots were generated using the smoothScatter function in the graphics package in R. For our plotting parameters, we used 250 bins for density estimation. The densities were represented (from least dense to most dense) by the following sequence of colors: white > beige > gray > black > orange > red.

### Nurses’ Health Study (NHS) cohort

The Nurses’ Health Study cohort was established in 1976 when 121,701 female US registered nurses ages 30 to 55 responded to a mail questionnaire that inquired about risk factors for breast cancer [26]. Every two years, women are sent a questionnaire and asked whether breast cancer has been diagnosed, and if so, the date of diagnosis. All women with reported breast cancers (or the next of kin if deceased) are contacted for permission to review their medical records so as to confirm the diagnosis. Pathology reports are also reviewed to obtain information on ER and PR status. Informed consent was obtained from each participant. This study was approved by the Committee on the Use of Human Subjects in Research at Brigham and Women’s Hospital.

### NHS tissue microarrays and immunohistochemistry

Tissue microarrays (TMAs) have been constructed from paraffin blocks of breast cancers that developed between 1976 and 2000 among women enrolled in the NHS. Details of TMA construction and IHC procedures for ER and PR have been previously described [27]. Briefly, immunohistochemical staining was performed for ER and PR on 5 μm paraffin sections cut from TMA blocks. Immunostains for each marker were performed in a single staining run on a Dako Autostainer (Dako Corporation, Carpinteria, CA, USA). The following antibodies and dilutions were used: for ER, a mouse monoclonal (clone 1D5) from Dako at 1:200 dilution; and for PR, a mouse monoclonal (PR 636) from Dako at 1:50 dilution. Study pathologists reviewed the immunostained sections under a microscope and estimated the percentage of tumor cells showing nuclear immunoreactivity in every tissue core. A case was considered as positive when there was staining in >1% of the tumor cell nuclei in any of the three cores from that case, and negative when no nuclear staining was seen in any of the three cores.

### Assessment of agreement between TMA- and medical record-based ER/PR classifications in NHS

A total of 2011 patients had information on ER and PR status from the medical record (MR) (28% by IHC, 72% by biochemical assays) and from TMAs (all by IHC). We computed the proportion of classifications in the MR that received concordant classifications by TMA and computed Kappa statistics for each of the four ER/PR subtypes (similar to the analysis in the GEM dataset). We note that in clinical practice the IHC cut-off for positive ER and PR staining changed from approximately 10% to 1% over the course of the study. This change may account for some inflation of the discordance estimates in the NHS dataset, as the cut-off of 1% was used for interpretation of the TMAs. We would expect this inflation to affect ER and PR similarly.

### Univariate and multivariate survival analyses in NHS

To assess the association of ER and PR expression with survival, we performed multivariate Cox regression to breast cancer-specific survival, using age, year of diagnosis, treatment, stage and grade as co-variates in the models.

## Results

### Progesterone receptor mRNA tends to be expressed at low-levels in ER- breast cancer and the ER-/PR+ subtype is extremely rare

**Figure 2:**
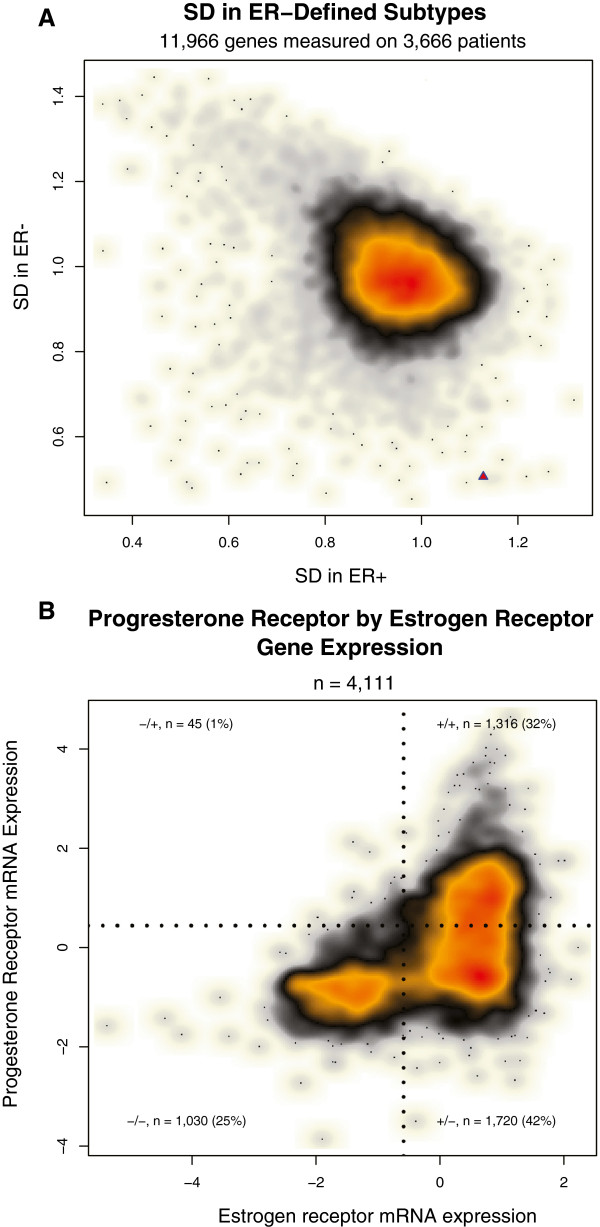
**Analyses of estrogen receptor and progesterone receptor mRNA expression in breast cancer. A**: Genome-wide analysis of expression variability in ER+ and ER- breast cancer. This smoothed scatterplot shows the distribution of 11,966 genes plotted based on their variability in mRNA levels in ER+ breast cancer (X axis) and ER- breast cancer (Y axis). The color represents the density of genes and ranges from white > beige > gray > black > orange > red, with red the most dense and white the most sparse. We computed the standard deviation (SD) of each gene within ER+ cases (n = 2,505) and ER- cases (n = 1,161). *PGR* is represented by a red triangle in the bottom-right portion of the plot, demonstrating that *PGR* shows highly variable expression in ER+ breast cancer (Ranked 157th out of 11,966 genes, 1.3th percentile). Conversely, *PGR* is one of the least variable genes in ER- breast cancer (Ranked 11,957th out of 11,966 genes, 99.9th percentile). **B**: Estrogen receptor and progesterone receptor mRNA expression in GEM dataset. This smoothed scatterplot shows the distribution of 4,111 breast tumors. Each tumor is plotted based on its *ESR1* expression level (X-Axis) and *PGR* expression level (Y-Axis). The color represents the data density and ranges from white > beige > gray > black > orange > red, with red the most dense and white the most sparse. The jagged black lines represent the cut-points for converting the continuous mRNA values into a positive/negative binary score. The cut-points used were −1.3 and 0.4 for *ESR1* and *PGR*, respectively. Based on these classification boundaries, 1,316 (32%) of cases were classified as ER+/PR+ (+/+), 1720 (42%) as ER+/PR- (+/−), 1,030 (25%) as ER-/PR- (−/−), and 45 (1%) as ER-/PR+ (−/+).

We performed a genome-wide analysis to determine the relative level of *PGR* expression and variability of *PGR* expression in ER- and ER+ breast cancer (Figure 2). To determine cut-points for ER and PR positivity based on the *ESR1* and *PGR* mRNA data, we fit a mixture of two Gaussians to the *ESR1* mRNA data and *PGR* mRNA data (separately), which produced a positivity cut-point of −1.3 for *ESR1* and 0.4 for *PGR*. Based on these cut-points, we classified each of 3,666 cancers as ER+ (2,505; 68%) or ER- (1,161; 32%) based on mRNA expression levels. We then computed the standard deviation of each gene separately in the ER+ and ER- cancers. This analysis demonstrates that *PGR*'s variability is strongly dependent on ER status (Figure 2A). *PGR* shows highly variable expression levels in ER+ breast cancer (*PGR* is more variable than approximately 98% of the genes in the genome among ER+ cancers). In contrast, *PGR* expression is highly invariable in ER- breast cancer (*PGR* expression is less variable than >99% of the genes in the genome in ER- breast cancer). These data are concordant with the observation that measurement of PR expression can be used to aid in the stratification of ER+ breast cancer into more- and less-aggressive disease subtypes [12,13,15]. The lack of variation of *PGR* expression in ER- breast cancer suggests that it is unlikely PR will provide clinically or biologically useful information for the stratification of ER- breast cancer.

To gain further insight into the relationship of *ESR1* and *PGR* expression, we performed a scatterplot of *ESR1* and *PGR* mRNA expression levels across 4,111 breast cancers (Figure 2B). This analysis shows that *ESR1* and *PGR* expression demonstrate a highly asymmetric relationship, in which *PGR* expression tends to be low/absent in ER- breast cancer, with >95% of ER- cases showing relatively low-levels of *PGR* expression (less than the cut-point of 0.4), while *PGR* expression varies from low-to-high in ER+ breast cancer, with 43% of ER+ breast cancers showing relatively high levels of *PGR* expression and 57% of ER+ breast cancers showing relatively low levels (Figure 2B). Thus, the ER-/PR+ subtype is by far the most rare (n = 45; 1%). All other ER/PR subtypes contain at least 25% of the cancers: ER+/PR+ (n = 1,316; 32%), ER+/PR- (n = 1,720; 42%), and ER-/PR- (n = 1,030; 25%).

**Figure 3:**
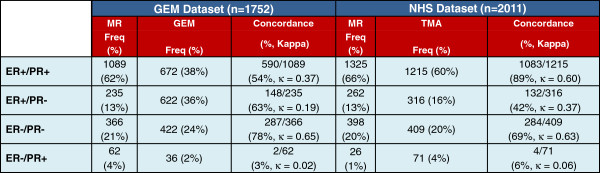
**ER and PR subtype frequency and inter-assay concordance.** MR, medical record; GEM, gene expression microarray; TMA, tissue microarray.

We assessed the ER/PR subtypes derived from the protein-based assays in the NHS and GEM datasets. The three protein-based analyses showed highly similar distributions of the ER/PR subtypes (Figure 3), with: 60 to 66% of cases classified as ER+/PR+, 13 to 16% as ER+/PR-, 20 to 21% as ER-/PR-, and only 1 to 4% as ER-/PR+. In general, the distributions of ER/PR subtypes were similar in the mRNA and protein-based analyses, with the exception of a significantly higher proportion of ER+ cases classified as PR- in the microarray data: approximately 50% of ER+ cases were classified as PR- in the mRNA dataset, compared with only approximately 20% in the protein expression data from the GEM dataset (*P* <2.2e-16) and 21% and 17% in the NHS MR and TMA protein-based analyses. In all analyses, the ER-/PR+ classification represented the rarest ER/PR subtype, accounting for between 1 to 4% of cases.

### ER-/PR+ is the least reproducible breast cancer subtype

**Figure 4:**
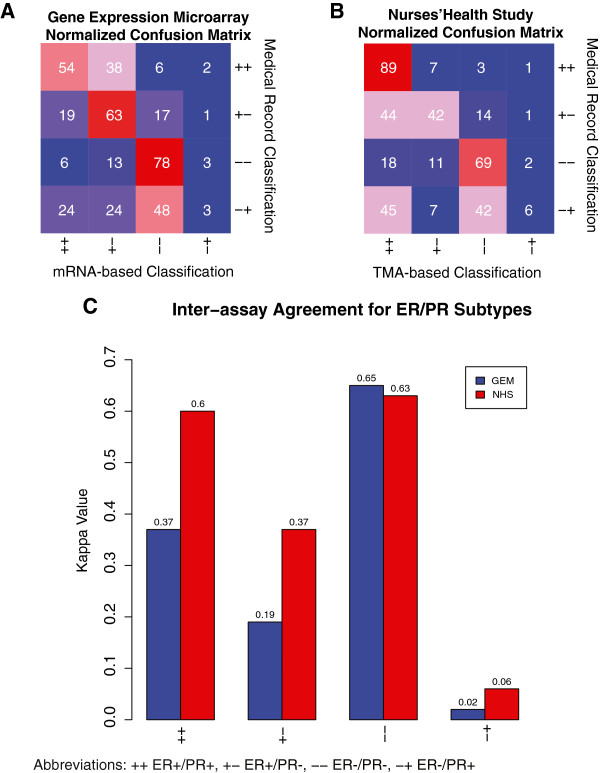
**Inter-assay agreement confusion matrices for ER/PR subtypes. A** and **B** present 4 × 4 confusion matrices. **A**: Gene Expression Microarray (GEM) Dataset. The row and columns indicate the ER/PR classifications made in the medical record from the GEM dataset (rows) and by mRNA (columns). The value in each cell in the matrix indicates the proportion of the row’s subtype that was classified in the column’s subtype. The color represents the proportion agreement from blue (low) to red (high). **B**: Nurses’ Health Study (NHS) Dataset. This confusion matrix is similar to that described in A, but the rows represent the ER/PR classifications from the medical record in the NHS dataset and the columns represent the classifications made from the NHS TMA analysis. **C**: Kappa Values for the gene expression microarray (GEM) and Nurses’ Health Study (NHS) datasets.

To gain further insight into whether ER-/PR+ breast cancer represents a true breast cancer disease subtype, we assessed the inter-assay reproducibility of ER/PR subtypes for cancers that underwent subtype classification by two methods (mRNA expression assessment by microarray vs. protein expression reported in the MR in the GEM dataset; and protein expression recorded in the MR vs. analyzed by IHC on TMAs in the NHS dataset). For each ER/PR subtype, we computed the proportion of cases in the MR that received the same classification by the second method, and we computed Kappa statistics for each ER/PR subtype (Figure 4).

For cases classified as ER+/PR+ by MR in the GEM dataset, 92% were classified as ER+ by GEM, although this percentage was split between ER+/PR+ (54%) and ER+/PR- (38%). In the NHS dataset, 89% of cases classified as ER+/PR+ by the MR received the same classification by TMA. The Kappa values for ER+/PR+ were 0.37 (95% CI 0.33 to 0.41) and 0.60 (95% CI 0.57 to 0.64) in the GEM and NHS datasets, respectively. As would be expected, we see greater inter-assay concordance in the NHS dataset, as both assays in the NHS dataset are protein-based, while the GEM dataset analyses are based on the agreement of protein and mRNA expression data.

For cases classified as ER+/PR- in the MR in the GEM dataset, 82% were classified as ER+ in the microarray data, with the ER+/PR- category the most common classification (63%). Similarly, in the NHS for cases classified as ER+/PR- in the MR, 86% were classified as ER+ in the TMA data with a relatively even split between ER+/PR+ and ER+/PR-. The Kappa values for ER+/PR- were 0.19 (95% CI 0.13 to 0.24) and 0.37 (95% CI 0.30 to 0.43) in the GEM and NHS datasets, respectively.

In the GEM dataset, 78% of ER-/PR- cases in the MR were classified as ER-/PR- by microarray. In the NHS dataset, 69% of ER-/PR- cases in the MR were classified as ER-/PR- in the TMA analysis. In both datasets, the majority of discordant cases were re-classified as ER+ by the second method (94% and 86% in the GEM and NHS datasets, respectively), with relatively few ER-/PR- cases reclassified as ER-/PR+. The Kappa values for ER-/PR- were 0.65 (95% CI 0.61 to 0.69) and 0.63 (95% CI 0.59 to 0.67) in the GEM and NHS datasets.

The ER-/PR+ category showed by far the lowest inter-assay agreement with concordance of only 2/62 (3%) and 4/71 (6%) of cases classified as ER-/PR+ in the MR in the GEM and NHS datasets, respectively. In both the GEM and NHS datasets, the ER-/PR+ cases were re-classified relatively evenly into ER+ and ER- subtypes, with a 50/50 and 55/45 split into ER+ and ER- subtypes in the GEM and NHS datasets, respectively. The Kappa values for ER-/PR+ were 0.02 (95% CI −0.18 to 0.21) and 0.06 (95% CI −0.12 to 0.25) in the GEM and NHS datasets, indicating no significant agreement (both 95% CIs include zero).

### ER classifications are more reproducible than PR classifications

To gain insight into the individual contributions of ER and PR to the reproducibility of joint ER/PR assessments, we assessed the inter-assay agreement of ER and PR separately. In the GEM dataset, there is a higher proportion of concordance for ER classifications as compared with PR: 1,526/1,752 (87%) agreement (Kappa = 0.66 (95% CI 0.62 to 0.70)) for ER classifications compared with 1,147/1,752 (65%) agreement (Kappa = 0.35 (95% CI 0.31 to 0.39)) for PR classifications (*P* for difference in proportions <2.2e-16). The NHS dataset shows similar findings, with more concordance in ER classifications as compared with PR (although the difference are smaller than seen in the mRNA vs. Protein analysis in the GEM dataset): 1,761/2,011 (88%) agreement (Kappa = 0.64 (95% CI 0.60 to 0.69)) for ER vs. 1,634/2,011 (81%) agreement (Kappa = 0.59 (95% CI 0.55 to 0.62)) for PR (*P* for difference in proportions = 4.3e-8).

We note that these Kappa estimates are likely underestimates of the inter-assay reproducibility observed in current clinical practice, since: 1) the GEM dataset-based analysis is comparing mRNA expression with IHC from data obtained across multiple different institutions; 2) protein expression data in the NHS MR were recorded by different laboratories, using multiple methods (IHC, biochemical assays), spanning several decades; and 3) the NHS TMA cases sampled only a subset of the tumor and did not have the benefit of the whole slide analysis used in routine clinical practice. Although these factors may produce an underestimate of Kappa values in our study, we would expect these limitations to affect the Kappa values for ER and PR relatively similarly, and thus, it is unlikely that these factors confound analyses of the relative reproducibility of ER compared with PR and of the relative distribution and relative reproducibility of the combined ER/PR subtypes.

### Progesterone receptor mRNA expression and breast cancer prognosis in ER-defined subtypes

**Figure 5:**
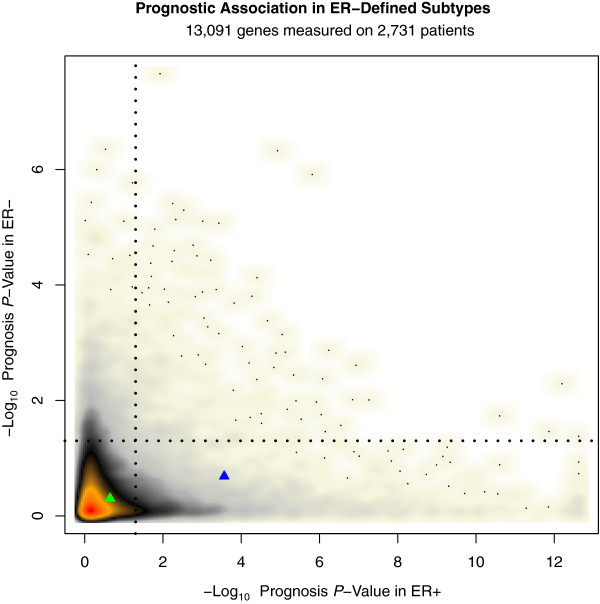
**Genome-wide survival analysis stratified by ER status.** This smoothed scatterplot shows the distribution of the prognostic association of 13,091 genes in ER+ (X-axis) and ER- (Y-axis) breast cancer. The *P*-values plotted have been corrected for multiple hypothesis testing using the method of Benjamini and Hochberg [25]. The color represents the density of genes and ranges from white > beige > gray > black > orange > red, with red the most dense and white the most sparse. The dotted black lines represent a significance threshold of adjusted *P* = 0.05. The blue triangle represents *PGR* and the green triangle represents *ESR1*. *PGR* expression is associated with prognosis in ER+ breast cancer; however, 727 genes are more prognostic than PR with the most prognostic genes showing a prognostic association to the significance level of *P* <1 × 10^12^ as compared with the prognostic significance level of 3 × 10^–4^ achieved by PR.

Next, we focused our analysis on progesterone receptor's prognostic association in ER+ and ER-negative breast cancer. *PGR* mRNA expression was significantly associated with improved prognosis in ER+ breast cancer (adjusted *P*-Value = 0.0003); however, in our genome-wide analysis, we identified hundreds of genes with stronger prognostic association in ER+ breast cancer (*PGR*'s association was ranked 728th out of the approximately 13 K genes (approximately 6th percentile), Figure 5, Additional file 1: Table S2). The set of genes more prognostic than *PGR* in ER+ breast cancer was highly enriched for genes associated with proliferation and cell cycle (for example, 12% of this set of genes was associated with the GO term mitotic cell cycle, false discovery rate (FDR) for enrichment = 3.4e-32), including the highly ranked gene *AURKA* (adjusted *P*-*valu*e <2.4e-13). In agreement with prior studies [13], we find that (in contrast to *PGR*) *ESR1* mRNA expression levels are not associated with survival in ER+ breast cancer (Figure 5).

Approximately 1.3 K genes were identified as significant at an adjusted *P*-value of 0.05 in ER- breast cancer. The set of top-ranked prognostic genes in ER- breast cancer was highly enriched for genes involved in the immune response (for example, 37% of the genes achieving an adjusted survival *P*-value of 1e-4 are associated with the GO term “immune response”, FDR for enrichment = 1.3e-11). *PGR* expression was not significantly associated with prognosis in ER- breast cancer (adjusted *P*-value = 0.21).

### Survival analyses incorporating ER and PR expression and clinico-pathologic factors

**Figure 6:**
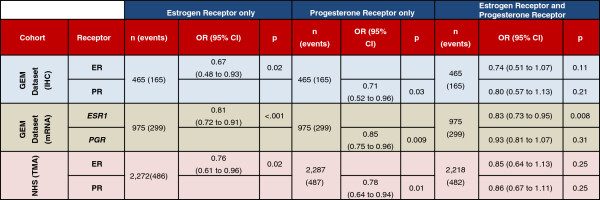
**Cox regression to overall survival.** The multivariate regression analyses to overall survival for the gene expression microarray (GEM) dataset are adjusted for nodal status, size, age and grade. Nurse’s Health Study (NHS) data are adjusted for age, year of diagnosis, treatment, stage and grade. Tumor size is measured in centimeters; nodal status is recorded as positive versus negative. IHC, immunohistochemistry; OR, odds ratio; TMA, tissue microarray.

To further evaluate the clinical significance of ER and PR expression, we built multivariate prognostic models incorporating ER and PR protein expression and standard clinico-pathologic factors. In the GEM dataset, a total of 465 patients had ER and PR protein expression data, covariate data and overall survival data available. When either ER or PR was included in multivariate prognostic models considering age, grade, tumor size (T) and nodal status (N), hormone receptor status was significantly associated with overall survival (Figure 6). When both ER and PR protein expression were included in the same multivariate prognostic model, neither ER nor PR made an independent contribution to the prognostic model.

We performed a similar set of analyses on the NHS dataset. To ensure consistent assessment of IHC staining, we used ER and PR as measured on the TMAs, as these were produced and interpreted at a central laboratory. Due to the different data points recorded for this cohort, age, treatment (chemotherapy and endocrine treatment, endocrine treatment only, chemotherapy only, or no treatment recorded), radiation (present vs. absent), stage and grade were included in multivariate models of breast cancer-specific survival. We found that, as with the protein expression data from the GEM dataset, ER and PR obtained statistically significant coefficients when included in separate multivariate Cox models, but neither was significant when both were included in the same model (Figure 6). To prevent any confounding of inclusion of endocrine treatment in the prognostic model considering ER and/or PR, we performed the analysis with the exclusion of the endocrine treatment covariate. We obtained highly similar results suggesting no significant confounding (Additional file 1: Table S4).

When our analyses were repeated using disease free survival (DFS) in the GEM dataset, ER by immunohistochemistry was significantly associated with DFS (*P* = .002) in a prognostic model considering age, grade, tumor size (T) and nodal status (N); however, PR was not (*P* = .151) when included in the model (without ER). When both hormone receptors by IHC were included in a model to DFS, neither obtained a significant coefficient (*P* = .67 for PR, .21 for ER), similar to results observed in the overall survival analyses (Additional file 1: Table S3). When using the mRNA data to DFS, neither of the hormone receptors achieved significant coefficients when either one or both were included in prognostic models. However, the GEM dataset was collected from multiple different institutions, and thus it is possible that different criteria were used to define DFS at different institutions, which may weaken the DFS analyses in this meta-dataset. On the NHS dataset, the DFS analysis was largely concordant with the results from the breast cancer-specific analysis (Additional file 1: Table S3), with significant (or borderline-significant) coefficients when ER and PR were included separately in a multivariate model, but non-significant coefficients when both were included in the same model (Additional file 1: Table S3).

Next, we evaluated the prognostic significance of combined hormone receptor status (ER+/PR+, ER+/PR-, ER-/PR-). Due to the extremely small sample size of ER-/PR+ cases and to the fact that the ER-/PR+ cases did not satisfy the proportional hazards assumption, we have excluded this classification from the combined hormonal receptor status multivariate survival analysis. We used the ER+/PR+ classification as our reference group. In both the GEM and NHS dataset, the ER+/PR- group showed no significant association with decreased survival as compared with the ER+/PR+ by IHC. By mRNA expression levels in the GEM dataset, the ER+/PR- group was associated with decreased survival (Additional file 1: Table S5).

## Discussion

It is recommended that all newly diagnosed breast cancers be evaluated for PR and ER protein expression by immunohistochemistry [1]. The clinical utility of ER as a predictive biomarker to identify breast cancer patients that will benefit from hormonal therapy is well established [14]. The added clinical value of assessing PR is controversial [2-4]. The goals of our study were to assess the frequency, reproducibility and prognostic association of breast cancer subtypes defined by ER/PR expression.

Prior work has shown that PR loss in ER+ breast cancer is associated with a more aggressive subset of ER+ breast cancer [9-13,15]. A limitation of most prior studies examining the prognostic significance of PR expression in ER+ breast cancer is that they have not examined the prognostic performance of PR relative to other genes, genome-wide. It has recently been shown that a large number of “randomly selected” genes and gene sets obtain statistically significant associations with patient prognosis in ER+ breast cancer [28], suggesting that prior to inferring the biological significance of a cancer biomarker (gene or gene signature) based on correlation with survival, it is necessary to determine the marker’s ability to stratify patients into prognostically variable groups relative to the performance of randomly selected genes/gene-sets in the dataset [24,28].

Our study contributes to the prior literature on the prognostic value of PR expression in breast cancer, by performing a genome-wide survival analysis of approximately 13 K genes across approximately 2.7 K patients stratified by ER status. In this analysis, *PGR* expression was associated with prognosis in ER+ but not in ER- breast cancer. However, *PGR* was not among the most strongly prognostic markers in ER+ breast cancer, ranking in the sixth percentile genome-wide, with approximately 5% of the approximately 13 K genes in the analysis showing at least as strong a prognostic association as *PGR* in ER+ breast cancer. Thus, in an unbiased genome-wide search for the most prognostic markers in ER+ breast cancer, progesterone receptor would be unlikely to be selected. In our multivariate survival analyses from both the GEM and NHS datasets, ER and PR were significantly associated with survival in multivariate survival models considering ER or PR and standard clinco-pathologic factors; however, when both hormone receptors were included in the same multivariate model, neither ER nor PR were significant.

The most important attribute of a cancer biomarker is not correlation with patient prognosis but efficacy in predicting response to specific therapies. It has long been hypothesized that evaluation of PR expression in ER+ breast cancer could be used to identify a patient subset most likely to benefit from hormonal therapy [7]. A recent meta-analysis of 20 randomized clinical trials of tamoxifen efficacy (n approximately 20 K) demonstrated that both ER+/PR+ and ER+/PR- patients show significant benefit from tamoxifen therapy, and PR is not a useful marker for predicting tamoxifen response in ER+ breast cancer [14]. A recent study evaluating the ability of PR expression to predict benefit from exemestane vs. tamoxifen in ER+ breast cancer similarly identified no association between PR expression and treatment benefit [29], providing further evidence to suggest that PR is a prognostic, but not a predictive biomarker in ER+ breast cancer [30]. The potential role of PR as a predictive biomarker for determining benefit from chemotherapy in ER+ breast cancer is also not well defined. A recent study by Viale *et al*. [31] assessed the added benefit of PR for predicting response to chemo-endocrine therapy in ER+ breast cancer, and the investigators did not identify a significant interaction of PR status with chemotherapeutic regimen in predicting disease free survival. The value of PR for predicting chemotherapy response in ER+ breast cancer remains uncertain, and this is an important area for future study.

The biological and clinical significance of PR expression in ER- breast cancer is poorly understood and is controversial [1,16,17]. Some studies have suggested that ER-/PR+ breast cancers show distinct clinical and biological features [9,15], implying that ER-/PR+ may represent a true breast cancer disease subtype. Other studies have maintained that ER-/PR+ breast cancer is too rare (0 to 0.1% frequency) to represent a true disease subtype and that as IHC-based methods for ER/PR assessment improve, the ER-/PR+ classification will become even rarer [2,17,18]. The recent EBCTG meta-analysis of randomized trials of tamoxifen efficacy identified a slight trend for PR expression to be associated with benefit from tamoxifen therapy in ER- breast cancer; although this result did not reach statistical significance [14].

Our study makes two primary contributions to the prior body of literature regarding ER-/PR+ breast cancer. First, we perform a large gene expression microarray-based analysis incorporating the measurement of mRNA levels of *ESR1* and *PGR* from approximately 4 K breast cancers. We find that *PGR* is one of the least variable genes in ER- breast cancer (ranked 10th genome-wide, <0.1 percentile), and the great majority of ER- cases show low/absent *PGR* expression levels. Thus, ER-/PR+ breast cancer is by far the most rare breast cancer subtype defined by ER/PR expression, accounting for approximately 1% of cases in the mRNA-based analyses. We find similar findings in the protein-based analyses, in which the ER-/PR+ subtype is the rarest ER/PR subtype, accounting for between 1% and 4% of the cases.

The consistency of the observation (both in our study, and in prior studies) that ER-/PR+ breast is by far the most rare breast cancer subtype, accounting for approximately 1 to 4% of cases, establishes that ER and PR show a highly asymmetric pattern of co-expression, in which ER- implies PR-, but PR- does not imply ER-. These “Boolean implications” [32] support the long-held biological model that PR is under the control of ER [5,6,8].

The second major contribution of our study to the characterization of ER-/PR+ breast cancer is that we performed an inter-assay reproducibility analysis across two large and diverse breast cancer datasets, in which ER and PR were assessed by multiple methods on the same set of tumors. This analysis shows that ER-/PR+ breast cancer is by far the least reproducible breast cancer subtype, with the vast majority (94% and 97% in the two datasets) of cases classified as ER-/PR+ in the MR re-classified when testing was performed by a secondary method. The re-classified cases were relatively evenly split between ER+ and ER- subgroups on repeat testing.

Taken together, our data do not support that ER-/PR+ represents a biologically distinct or clinically useful breast cancer subtype. These data suggest that PR testing is not warranted in ER- breast cancer, as ER-/PR+ breast cancer is very rare and non-reproducible, thus the vast majority of cases classified as ER-/PR+ will represent false classifications. Our data suggest that ER+/PR- breast cancer represents a distinct disease subtype, which accounts for approximately 15% of breast cancers, shows fair reproducibility, and is associated with worse prognosis as compared with ER+/PR+ breast cancer; however, our genome-wide analysis identifies hundreds of genes that are significantly more prognostic than PR in ER+ breast cancer, suggesting that other candidate prognostic biomarkers are likely to outperform PR for predicting patient survival in ER+ breast cancer. Further, until there are data to establish that PR is a predictive (and not merely prognostic) marker in ER+ breast cancer (and outperforms competing predictive biomarkers in ER+ breast cancer), the clinical rationale for routine PR testing in ER+ breast cancer will remain uncertain.

## Conclusions

The College of American Pathologists and American Society of Clinical Oncology recommend ER and PR testing for all newly diagnosed cases of invasive breast cancer [1]. While the clinical and biological importance of ER in breast cancer is well-established, the added clinical benefit of PR evaluation is uncertain. In our integrative analysis, incorporating gene expression profiling data, immunohistochemistry data, and clinical data across two large and diverse datasets, we find that:

1. PR tends to be expressed at low levels in ER- breast cancer.

2. PR expression is not associated with prognosis in ER- breast cancer.

3. ER-/PR+ breast cancer is not a reproducible subtype.

Thus, PR testing is of uncertain clinical utility in ER- breast cancer. The clinical utility of measuring PR expression in ER+ breast cancer is also not well-defined. Several studies (including ours) show that loss of PR expression is associated with a more aggressive subset of ER+ breast cancer; however, it is important to note that testing for PR expression currently provides no clinically actionable information in ER+ breast cancer, as patients will receive endocrine therapy regardless of PR status and there is no consensus as to whether knowledge of PR expression by IHC has a role in informing the use of chemotherapy in ER+ breast cancer. Further, our study identifies hundreds of genes that are more prognostic than PR in ER+ breast cancer demonstrating that it is unlikely that PR will emerge as a top-performing prognostic biomarker in ER+ breast cancer. Therefore, there is currently no strong evidence to support the clinical utility of routine PR testing in ER+ or ER- breast cancer. Given that breast cancer is the most common cancer diagnosed in women, eliminating PR testing from the routine diagnostic work-up of invasive breast cancer could save the health care industry tens of millions of dollars per year, with no loss in the clinical utility of the pathological evaluation.

## Abbreviations

DFS: Disease free survival; EBCTCG: Early Breast Cancer Trialists’ Collaborative Group; ER: Estrogen receptor; ESR1: Estrogen receptor mRNA; FDR: False discovery rate; GEM: Gene expression microarray; IHC: Immunohistochemistry; MR: Medical record; NHS: Nurses’ health study; PGR: Progesterone receptor mRNA; PR: Progesterone receptor; SEER cancer registry: Surveillance, epidemiology, and end results; TCGA: The cancer genome atlas; TMA: Tissue microarray.

## Competing interests

AHB is a consultant to Roche Diagnostics and is supported by an award from the Klarman Family Foundation. This work was supported by GlaxoSmithKline (WE234 (EPI40307)) and a Public Health Service Grant CA087969 from the National Cancer Institute, National Institutes of Health, Department of Health and Human Services.

## Authors' contributions

MMH participated in the statistical analyses and wrote the manuscript. RH and NWK participated in the statistical analyses. RMT and LCC participated in the study design and helped to draft the manuscript. BHK helped to coordinate the gene expression dataset and helped to draft the manuscript. AHB participated in study design, statistical analyses, and wrote the manuscript. All authors read and approved the final manuscript.

## Supplementary Material

Additional file 1: Table S1 Description of gene expression microarray datasets used in the analyses. **Table S2.** Results of genome-wide survival analysis stratified by ER status. Columns 3 and 4 present the Wald-test statistic for prognostic association in ER- and ER+ breast cancer, respectively. Columns 5 and 6 present the adjusted *P*-value for the prognostic association in ER- and ER+ breast cancer, respectively. Columns 7 and 8 present the negative log_10_ of the adjusted *P*-values (these values are plotted in Figure 5). **Table S3.** Disease-free survival analysis. **Table S4.** NHS survival analysis including and excluding endocrine therapy. **Table S5.** Survival analysis with combined receptor status. Click here for file
